# Nonocclusive Mesenteric Ischemia Rescued by Immediate Surgical Exploration in a Boy with Severe Neurodevelopmental Disability

**DOI:** 10.1155/2019/5354074

**Published:** 2019-02-19

**Authors:** Manami Mizumoto, Fumihiro Ochi, Toshihiro Jogamoto, Kentaro Okamoto, Mitsumasa Fukuda, Toshifumi Yamauchi, Toyohisa Miyata, Ryo Tashiro, Mariko Eguchi, Riko Kitazawa, Eiichi Ishii

**Affiliations:** ^1^Department of Pediatrics, Ehime University Graduate School of Medicine, Shitsukawa, Toon, Ehime 791-0295, Japan; ^2^Division of Diagnostic Pathology, Ehime University Graduate School of Medicine, Shitsukawa, Toon, Ehime 791-0295, Japan

## Abstract

**Background:**

Nonocclusive mesenteric ischemia (NOMI) defines acute mesenteric ischemia without occlusion of the mesenteric arteries. The most common cause of NOMI is vasoconstriction or vasospasm of a mesenteric artery. NOMI generally affects patients >50 years of age, and few cases have been reported in children.

**Case Presentation:**

A 15-year-old boy with severe neurodevelopmental disability developed sudden-onset fever, abdominal distention, and dyspnea. Laboratory and radiological findings indicated acute intestinal obstruction and prerenal failure. He developed transient cardiopulmonary arrest and hypovolemic shock. Emergent laparotomy was performed, which revealed segmentally necrotic intestine from the jejunum to the ascending colon with pulsation of peripheral intestinal arteries, leading to a diagnosis of NOMI. The necrotic intestine was resected, and stomas were created. He was discharged on postoperative day 334 with short bowel syndrome as a complication.

**Conclusions:**

NOMI should be considered a differential diagnosis for intestinal symptoms with severe general conditions in both adults and children with underlying disease. Immediate surgical exploration is essential with NOMI to save a patient's life.

## 1. Introduction

Nonocclusive mesenteric ischemia (NOMI) defines acute serious mesenteric ischemia without occlusion of the mesenteric arteries. The prognosis of NOMI is poor, and the mortality rate is from 50% to 80% [1]. The most common cause of NOMI is vasoconstriction or vasospasm of a mesenteric artery. NOMI generally occurs in adults, especially in patients older than 50 years of age, and few cases have been reported in children.

Children with severe neurodevelopmental disability frequently experience gastrointestinal problems, most commonly, chronic constipation. Factors contributing to chronic constipation include prolonged immobility, skeletal abnormalities, extensor spasm, generalized hypotonia, and abnormal intestinal motility associated with certain neurological lesions [2]. However, few reports discuss NOMI in children with severe neurodevelopmental disability.

Early diagnosis and prompt treatment are essential for survival in patients with NOMI although nonspecific clinical signs and symptoms make the diagnosis extremely difficult to confirm. Although conventional angiography is considered the gold standard imaging method in patients with acute mesenteric ischemia [1, 3], many patients cannot tolerate angiography because of their unstable systemic conditions. Surgery is useful not only to resect persistently ischemic or necrotic intestine but also to evaluate intestinal viability to make a correct diagnosis [3, 4].

## 2. Case Presentation

A 15-year-old boy with severe neurodevelopmental disabilities developed sudden-onset fever, abdominal distention, and dyspnea and was referred to our hospital 3 hours after symptom onset. He was born at 36 weeks of gestational age by normal spontaneous vaginal delivery with an Apgar score of 8/10 and birth weight of 1836 g. He had multiple malformations and had undergone several surgeries including Ramstedt pyloromyotomy, ventricular septal defect repair, orchidopexy, Nissen fundoplication, and scoliosis correction. Conventional G-banding analysis of his peripheral blood showed a normal karyotype.

He was taking several medications including magnesium oxide, daikenchuto (a Japanese herbal medicine), dimethicone, mosapride, and lansoprazole for gastrointestinal symptom control. Nitrazepam, risperidone, and carbamazepine were prescribed for insomnia and epilepsy. He had severe intellectual impairment and limited activities of daily living. He walked with a body support walker and had difficulty with oral feeding and no verbal communication ability.

**Figure 1:**
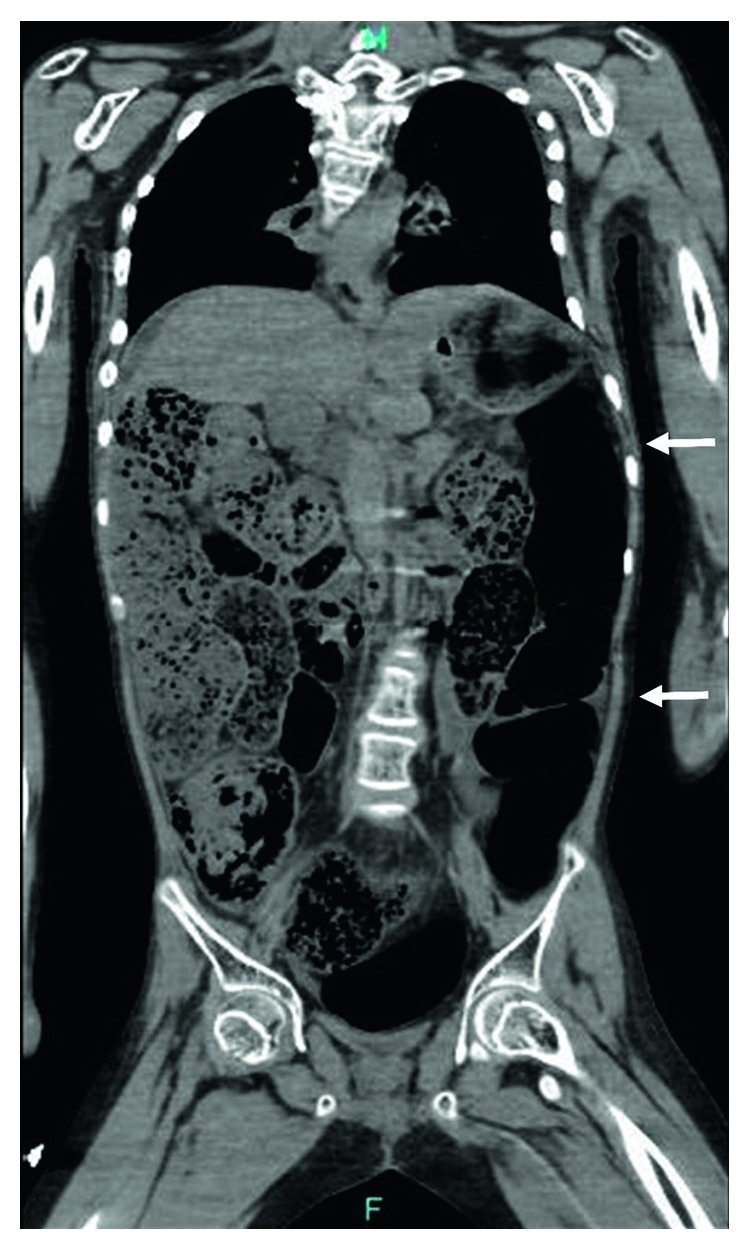
Abdominal computed tomographic image. The arrows show the patient's dilated and thinned intestines from the duodenum to the sigmoid colon.

On admission, he appeared unwell; his body temperature was 38.7°C, pulse rate was 110/min, and respiratory rate was 24/min. Physical examination revealed no abdominal distension or tenderness. Laboratory examination showed a normal white blood cell count with a slightly increased C-reactive protein level and normal serum levels of bilirubin, ammonia, glucose, and lactate. Serum levels of potassium, sodium, and chloride were 5.2 mEq/L, 134 mEq/L, and 102 mEq/L, respectively. Blood urea nitrogen and serum creatinine were 10 mg/dL and 1.41 mg/dL, respectively. Serum levels of aspartate aminotransferase, alanine aminotransferase, and lactate dehydrogenase were elevated at 83 U/L, 41 U/L, and 475 U/L, respectively. The platelet count was normal at 34.3 × 10^4^/*μ*L. The plasma fibrinogen level and antithrombin III level were 237 mg/dL and 69.3%, respectively. Prothrombin time expressed as internal normalized ratio (PT-INR) was 1.24, and activated partial thromboplastin time was 33.2 sec. Abdominal computed tomography (CT) without contrast revealed dilated and thinned intestinal loops from the duodenum to the sigmoid colon, without free air or fluid (Figure 1).

**Figure 2:**
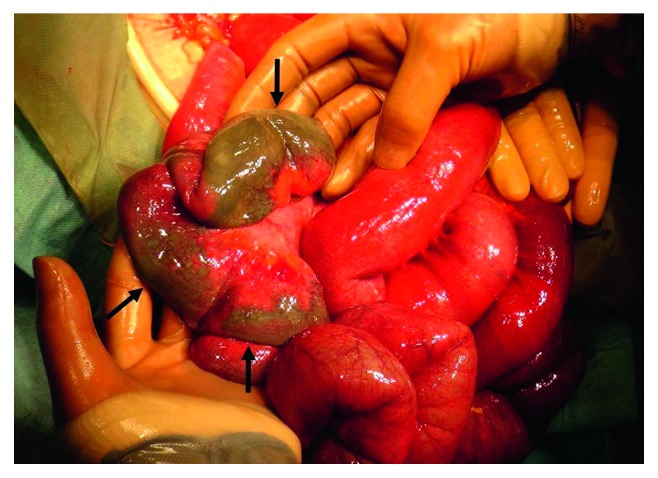
Intraoperative photograph. The arrows indicate the segmentally necrotic ascending colon.

**Figure 3:**
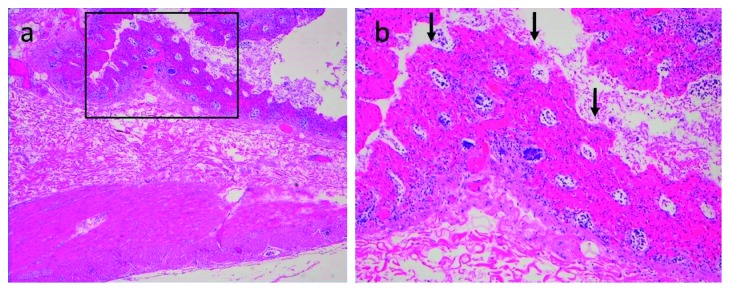
Histopathological findings of the colonic wall. (a) 20x magnification. (b) 100x magnification (inset). Transmural ischemic necrosis with hemorrhages is indicated by the arrows (hematoxylin and eosin stain).

The patient was initially diagnosed as having paralytic ileus secondary to severe constipation. He received nasogastric suction and intravenous fluids, and his dyspnea subsequently resolved. We had discussed further examinations including contrast CT when his condition suddenly worsened to shock presenting with ventricular tachycardia and absent distal pulses, cool extremities, and prolonged capillary refill. After appropriate cardiopulmonary resuscitation, he was transferred to an intensive care unit. The ventricular tachycardia diminished without electrical defibrillation. Isotonic crystalloids, albumin solution, dopamine, and dobutamine were administrated under the diagnosis of hypovolemic shock. Meropenem and intravenous immunoglobulin were also given for a provisional diagnosis of sepsis from bacterial translocation. It is considered that the patient developed disseminated intravascular coagulation (DIC) because of a decreased platelet count of 19.1 × 10^4^/*μ*L, decreased fibrinogen level of 109 mg/dL, decreased antithrombin III level of 41.8%, and prolonged PT-INR and APTT of 1.64 and 51.0 sec, respectively. The D-dimer level was 88.0 *μ*g/mL, and the thrombin-antithrombin complex level was 42.1 *μ*g/L, which were also consistent of DIC. Recombinant human soluble thrombomodulin and antithrombin III were used for DIC; however, these treatments were ineffective. The serum potassium level was 4.4 mEq/L, and other electrolyte levels were within normal limits. The serum amylase level was 351 U/L. We did not perform blood gas analysis that time because of some technical problems. Fifteen hours after admission, emergent laparotomy was performed because colonic obstruction secondary to fecal impaction was suspected. Surgery revealed a segmentally necrotic intestine from the jejunum to ascending colon with unattenuated pulsation of peripheral intestinal arteries (Figure 2), suggesting a diagnosis of NOMI. The necrotic intestine was resected, and stomas were created. Histopathology of resected intestine showed massive cellular infiltration, which was mainly neutrophilic, and ischemic changes with transmural necrosis (Figure 3). Arterial or venous thrombus was not identified in the resected specimens, which also supported the diagnosis of NOMI. On postoperative day 3, subserosal hemorrhage of the sutured jejunum occurred, which required surgical resection of the affected section. Extubation was difficult because of aspiration pneumonia; therefore, laryngotracheal separation was performed. He developed short bowel syndrome postoperatively and needed long-term supportive care. He was discharged on postoperative day 334.

## 3. Discussion

Although no specific marker indicating the onset of NOMI has been defined, we considered ventricular tachycardia a direct trigger in our patient. It is also possible that severe gastrointestinal symptoms before admission induced NOMI. NOMI is usually caused by splanchnic hypoperfusion resulting from a variety of conditions, including myocardial failure, septic shock, high-dose vasopressor infusions, hemodialysis, and advanced age [5, 6].

NOMI generally occurs in patients over 50 years of age. NOMI is rare in children but have been described in several reports. Marinez et al. report two children cases with NOMI resulting from burns where propranolol was used to reduce the hypermetabolic burn state, along with hypovolemic or septic shock, which ended in death despite intensive care and surgery [7]. An 11-year-old boy with Addison's disease and related shock [8] and an 18-year-old girl with acute lymphoblastic leukemia receiving chemotherapy [9] were also reported. It is notable that most of the reported patients, including our patient, had underlying diseases, which might trigger colonic complications.

NOMI is a rare condition, but the possibility must be considered because delayed diagnosis leads to high mortality [6]. Angiography is the gold standard for correct diagnosis [1]; however, NOMI often occurs in ill, unstable patients where angiography may not be possible because of its complexity and invasiveness. Abdominal CT with contrast may reveal portal venous gas or pneumatosis intestinalis, but these findings are nonspecific and found in a variety of conditions [5]. Therefore, abdominal CT has limited usefulness in the diagnosis of NOMI. In fact, our patient was initially diagnosed as having paralytic ileus secondary to severe constipation based on abdominal CT. Considering these issues, surgical exploration is currently the only tool providing an accurate assessment of intestinal viability and delineating frankly necrotic sections requiring segmental resection. We should not hesitate to use a surgical procedure when NOMI is suspected.

In conclusion, NOMI should be considered a differential diagnosis for intestinal symptoms in both adults and children with severe general conditions and underlying disease. Early surgery is the only practical way to diagnose NOMI and save patients' lives.

## Abbreviations

NOMI: Nonocclusive mesenteric ischemia
CT: Computed tomography.
